# Periodontitis induced by orthodontic wire ligature drives oral microflora dysbiosis and aggravates alveolar bone loss in an improved murine model

**DOI:** 10.3389/fmicb.2022.875091

**Published:** 2022-09-08

**Authors:** Rongshuang Ai, Dingyi Li, Luyao Shi, Xiaonan Zhang, Zhiqiang Ding, Yiting Zhu, Yujuan He

**Affiliations:** ^1^Key Laboratory of Diagnostic Medicine (Ministry of Education), Department of Laboratory Medicine, Chongqing Medical University, Chongqing, China; ^2^College of Stomatology, Chongqing Medical University, Chongqing, China; ^3^School of Computer Science, Chongqing Institute of Engineering, Chongqing, China; ^4^Department of Laboratory Medicine, Chongqing University Three Gorges Hospital, Chongqing, China

**Keywords:** orthodontic wire, ligation, inflammation, alveolar bone loss, 16S rRNA, microbiota dysbiosis

## Abstract

**Aim:**

To assess the contribution of polymicrobial disruption of host homeostasis to periodontitis progression in orthodontic wire ligation murine model.

**Methods:**

Orthodontic wire rings were inserted between the first and second molars of mice for 18 days for the orthodontic wire ligation mouse model, and *Pg* injection model and *Pg*-LPS injection model were used as controls. Alveolar bone loss and periodontal inflammation were analyzed by micro-CT, histological staining and qRT-PCR. Further, pyrosequencing of 16S rRNA gene amplicon was used to analyze the development of oral microorganism dysbiosis in the mice.

**Results:**

Micro-CT, TRAP staining and qRT-PCR showed that orthodontic wire ligation model led to more severe alveolar bone loss than *Pg* and *Pg*-LPS models.

H&E staining and qRT-PCR demonstrated that stronger inflammatory response was induced by the orthodontic wire treatment compared to the other models. In addition, pyrosequencing of 16S rRNA gene amplicons revealed that the composition of oral microbiota presented a transition as the disease progressed and significant differences emerged in oral microbiota communities between orthodontic ligature mice and healthy controls. Furthermore, antibiotic treatment decreased both inflammation and alveolar bone loss in response to microbial community dysbiosis. However, no significant difference in bacterial community composition was observed in *Pg* and *Pg*-LPS models.

**Conclusions:**

Orthodontic wire ligation drove oral microbial community transitions that mimicked polymicrobial communities characterized by polymicrobial synergy and dysbiosis. Our improved model is suitable for further study of pathogenesis of periodontitis and exploration of corresponding treatment strategies.

## Introduction

Periodontitis is a chronic inflammatory disease initiated by dysbiotic bacterial communities forming biofilms that leads to destruction of supportive tissues of teeth. More than 600 bacterial species are associated with tissues in the human oral cavity (Dewhirst et al., 2010), and periodontitis is specifically correlated with gram-negative anaerobic bacteria at the symptom site (Paster et al., 2001). Although thought to be linked to a defined microbial composition found on the surface of the tooth and tooth root, the contribution of polymicrobial disruption of host homeostasis to periodontitis progression remains poorly defined.

The development of periodontitis includes four discrete phases: (1) development of a pathogenic biofilm, (2) stimulation and invasion by oral microorganisms or their derived products, (3) induction of a destructive host response in gingival tissue, and (4) breakdown of the supporting tissue and alveolar bone. At present, several models are commonly used to mimic the different pathogenic phases of periodontitis, including silk ligation model, oral inoculation model and gingival injection model. The silk ligation model allows analysis of most aspects of periodontitis, including bacterial interactions and dysbiosis, periodontal inflammatory responses and bone biology, and hence has been recognized as the most suitable model for investigating the interaction between oral microorganisms and host responses during periodontitis development (Abe and Hajishengallis, 2013). In this model, inserting ligature threads is the key step, which is easy to achieve in large animals, like rats, rabbits, dogs and non-human primates, but difficult in mice due to the considerably limited operating space. In the oral inoculation model (de Molon et al., 2020; Hamamoto et al., 2020), periodontopathogenic bacteria (i.e., *Porphyromonas gingivalis, Pg*) is inoculated orally. In the gingival injection model (Akkouch et al., 2019; Leira et al., 2019), *Porphyromonas gingivalis* lipopolysaccharide (*Pg*-LPS) or *Pg* is injected into gingiva to initiate periodontitis. In contrast with silk ligation model, the latter two are more easy to achieve in mice. However, oral *Pg* inoculation model has been reported that wild-type C57BL/6J mice are less susceptible to alveolar bone loss (de Molon et al., 2020), and gingival *Pg*-LPS injection model is not considered to be able to mimic oral microflora dysbiosis. To this end, it is necessary to develop a more simple and economical ligature-induced periodontitis model to overcome the technical challenge of the traditional ligature placement in mouse gingival tissue.

In recent 10 years, orthodontic wire, a common tool in the dental clinic, was selected to replace silk to establish the mouse ligation model (Okamoto et al., 2009; Mizuno et al., 2015; Suzuki et al., 2016; Li et al., 2020). A stainless steel orthodontic wire was placed into the interproximal spaces between the molars (M1-M2), initiating periodontal inflammatory responses and alveolar bone loss. However, this method ignores several noteworthy problems, including oral microflora dysbiosis, ligation wire shedding, and/or mucosal tissue damage. In this study, we modified the ligating method of orthodontic wire, completely avoiding the loss of ligature and mucosal tissue damage. More importantly, similar to silk ligation model, periodontitis induced by orthodontic wire ligature drives oral microflora dysbiosis, periodontal inflammatory response and then aggravates alveolar bone loss in mice model.

## Materials and methods

### Mice

Wild-type female C57BL/6 mice aged 8–10 weeks were obtained from Chongqing Medical University. All mice were housed in a specific-pathogen-free environment. All procedures involving animals were performed in accordance with the Institutional Animal Care and Use Committee's guidelines at Chongqing Medical University and comply with the ARRIVE Checklist (Percie du Sert et al., 2020) (see more details in the Appendix).

### Mouse model of periodontitis

The mouse periodontitis models were established using *Pg, Pg*-LPS injection and orthodontic wire ligature separately. Each group contained 5 C57BL/6 mice. Mice were anesthetized with an intraperitoneal injection of 1.5% pentobarbital sodium (0.1 mL/20 g). For the orthodontic wire ligature model, a 0.2 mm stainless steel orthodontic wire was inserted into the gap between the first molar (M1) and second molar (M2). The procedure was performed as follows (Appendix Figure 1). As the mouse approached full anesthetization (3–5 min), expose the maxillary in a fixed supine position. (2) Held a 7- to 10-inch length of sterile orthodontic wire in your right hand and insert it from palatal to the buccal side into the gap between M1 and M2. To avoid bleeding, the orthodontic wire should not be inserted too deeply. (3) Held the tip of the orthodontic wire on the buccal side with a bending forcep and bent toward the opposite side to anastomose with the orthodontic wire on the palatal side to form a ring to prevent the wire from falling off. (4) Cut the redundant orthodontic wire with crown scissors and adjusted the closure of the orthodontic wire ring to avoid scratching the oral mucosa. The whole procedure, taking about 10 minutes to complete, should be done gently and carefully. In the *Pg* and *Pg*-LPS models, 1 μL of *Porphyromonas gingivalis* (W83) suspensions (1 × 10^9^ CFUs/mL in PBS) or 1 μL of *Pg*-LPS (1 μg/μL, Standard, InvivoGen, San Diego, CA) was injected into the gingival sulcus of the second maxillary molar (M2) using with a microsyringe (10 μL) once every 3 days for 18 days. The NC control mice received the same surgery but without the orthodontic wire placement, PG injection or PG-LPS injection. Mice in each group were randomly sacrificed at days 6, 12, and 18 to collect tissue samples for subsequent experiments. *Pg* were inoculated on Columbia sheep blood agar plates for 48–72 h at 37°C in <0.1% O_2_ and 7–15% CO_2_.

### Bacterial load determinations

Oral cavities of anesthetized mice were swabbed for 30s using sterile ultra-fine swabs. The bacteria on the swab were fully dissolved in 500 μL sterile PBS. The samples were serially diluted and plated on blood agar and cultured under anaerobic and aerobic conditions at 37°C for enumeration of bacterial loads. Anaerobic culture bags were purchased from Pangtong (Chongqing, China).

### Microbiota analysis

The oral cavities of anesthetized individual mice were swabbed for 30 s, and DNA was extracted from the swabs using the UltraClean Tissue and Cells DNA Isolation Kit according to the manufacturer's instructions (Takara, Japan). After confirming high DNA purity, the DNA samples were analyzed by qRT-PCR. In order to examine bacterial diversity, specific primers were designed against the r16S genes of certain bacterial phyla: these primers were used for qRT-PCR analysis, and the relative abundance of each phylum from total r16S was calculated. The following primers were used: *Bacteroidata*, 5′-ACGCACGGGTGAGTAACACGTAT-3′ and 5′-AGGGGATAAATCCTCTCAGTTCCCCT-3′; *Firmicutes*, 5′-GGAGTATGT GGTTTAATTCGAAGCA-3′ and 5′-AGCTGACGACAACCATGCAC-3′; *Proteobacteria*, 5′-GCTGATCATCCTCTCAGACAA-3′ and 5′-AGAACGAACGCTGGCGTAA-3′; *Actinobacteria*, 5′-GCTGATCTGCGATTACTAGCGTC-3′ and 5′-ATGTCTTGGGCTTCACGCA-3′; Universal, 5′-FAAACTCAAAKGAATTGACGG-3′ and 5′-CTCACRRCACGACCTGAC-3′. The specific primers for other target genes were described in Table 1.

**Table 1:** Primer sequences for amplification of mouse gene.

**Gene**	**Primer sequences**
*Il1β*	Sense primer: 5′-TCGCAGCAGCACATCAACAAGAG-3′ Anti-sense primer: 5′-AGGTCCACGGGAAAGACACAGG-3′
*Tnf-α*	Sense primer: 5′-GCCTCTTCTCATTCCTGCTTGTGG-3′ Anti-sense primer: 5′-GTGGTTTGTGAGTGTGAGGGTCTG-3′
*Il6*	Sense primer: 5′-CTTCTTGGGACTGATGCTGGTGAC-3′ Anti-sense primer: 5′-AGGTCTGTTGGGAGTGGTATCCTC-3′
*RunX2*	Sense primer: 5′-TCCCAGGCAGGCACAGTCTTC-3′ Anti-sense primer: 5′-AGCGGCGTGGTGGAGTGG-3
*Alp*	Sense primer:5′-CACGGCGTCCATGAGCAGAAC-3′ Anti-sense primer: 5′-CAGGCACAGTGGTCAAGGTTGG-3
*RANKL*	Sense primer:5′-ATGGAAGGCTCATGGTTGGATGTG-3′ Anti-sense primer: 5′-TGGCAGCATTGATGGTGAGGTG-3′
*Opg*	Sense primer:5′-TGCACAGTGAGGAGGAAGACATTG-3′ Anti-sense primer: 5′-CACTCCTGCTTCACGGACTGC-3′
*Ocn*	Sense primer:5′-AAGCCTTCATGTCCAAGCAGGAG-3′ Anti-sense primer: 5′-CGGTCTTCAAGCCATACTGGTCTG-3′
*Gapdh*	Sense primer: 5′-TGGAAAGCTGGGCGTGATG-3′ Anti-sense primer:5′-TACTTGGCAGGTTTCTCCAGG-3′

### Micro-CT

The entire maxillary of the mice were removed and scanned and analyzed using a VivaCT40Micro-CT system (Sanco Medical AG, Switzerland). The rebuilt images of bone surfaces were used to perform three dimensional histomorphometric analyses using the same density.

### TRAP staining

Histochemical markers of osteoclasts were stained with TRAP by using a leukocyte-specific acid phosphatase kit in accordance with the producer's protocol (Solarbio, Beijing, China). TRAP-stained sections of gingival tissues harvested at each time point after orthodontic wire placement were display at low (×40 magnification; scale bar, 0.5 mm) and high (×100 magnification; scale bar, 200 μm) magnifications. Arrowheads mark osteoclasts. The number of osteoclasts per square millimeter was calculated based on the number of osteoclasts per slide and the area of each slide.

### Bone loss measurements

The whole maxillary bone from our experimental mice was removed and boiled in water for 10 min. The attached muscle and tendons were removed and the maxillae were brushed, bleached and stained using 1 % methylene blue. Images of the maxillae were captured using a Perfection V200 Photo scanner (Epson, Japan). The distance on the distal side of M1 and mesial side of M2 on each of the buccal surfaces and palatal surfaces between the cementoenamel junction and alveolar bone crest (CEJ-ABC distance) was measured using image J (https://imagej.nih.gov/ij/).

### Histological analysis

The fixed maxillae were decalcified in a solution of 10% EDTA (pH 8.0) for 4 weeks at 4°C (EDTA solution was changed four times during this period). The decalcified tissue was embedded in paraffin with the buccal side of the tooth facing toward the bottom of the micro mold and the long axis of the teeth paralleling the short side of the cassette, sliced at 7 μm in the sagittal direction along the long axis of the teeth. After that, they were stained with hemotoxylin and eosin (HE) and digitally recorded with Nikon ECLIPSE 80i. The infiltration of inflammatory cells was calculated based on area invaded by inflammatory cells per slide and the area of each slide.

### Antibiotic treatment

Mice were treated with 1 mg/ml ampicillin and 1 mg/ml metronidazole in drinking water from 2 weeks before the orthodontic wire placement and throughout the modeling.

### Statistical analyses

Statistical analysis was performed using GraphPad Prism version 5.0 software (GraphPad, Software, San Diego, CA, USA) and data are presented as the mean ± SD. The Student's *t*-test was used to determine differences between two groups. One-way ANOVA followed by Bonferroni's *post hoc* test was used for multiple group comparisons. All experiments were carried out at least three times. *P* <0.05 indicated a statistically significant difference.

## Results

### Characterization of alveolar bone loss and osteoclast activation

**Figure 1:**
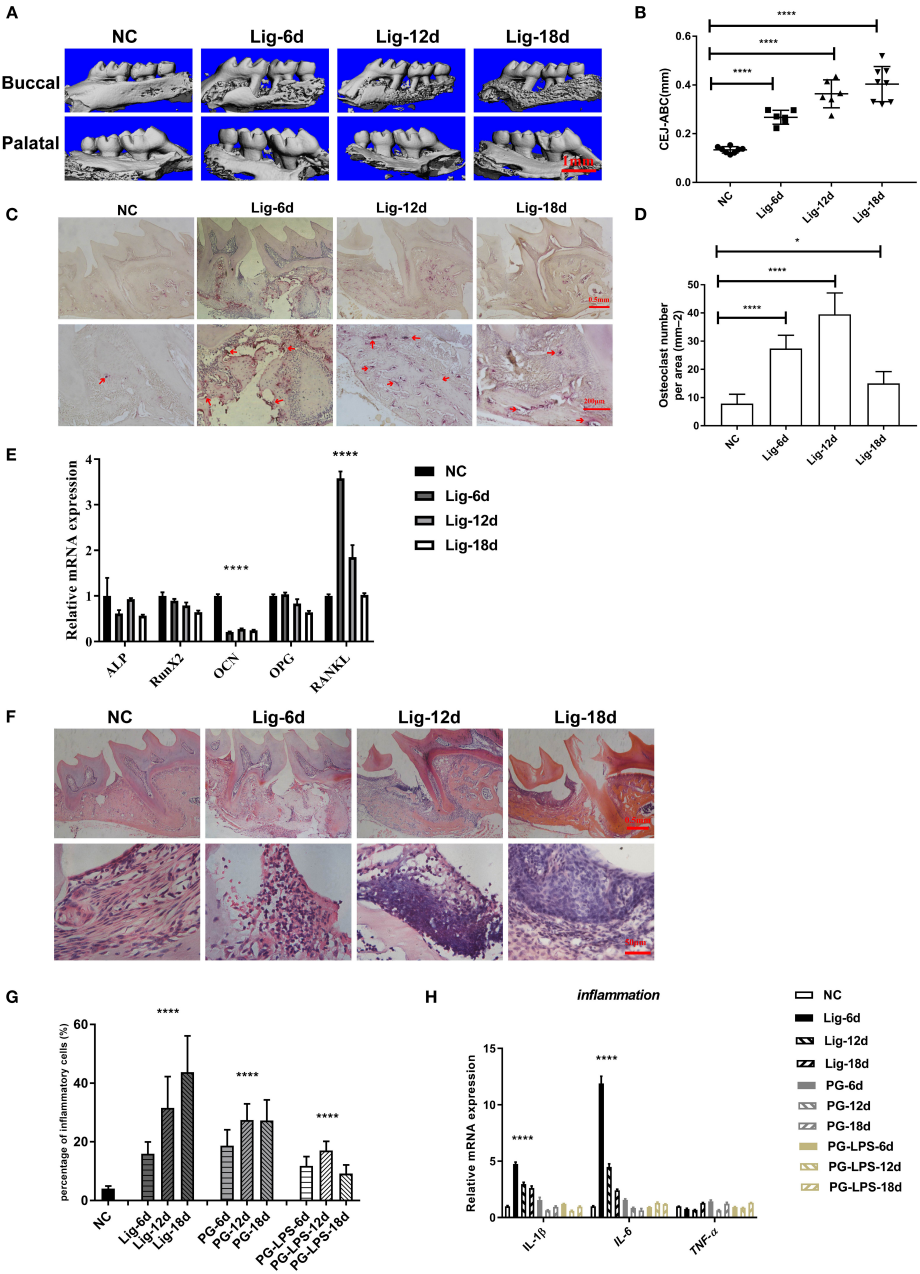
Orthodontic wire ligature induces progressive alveolar bone resorption and inflammatory response. **(A)** Representative sagittal 3D images viewed from the buccal side and palatal side of the maxillary molars using Micro-CT, scale bar, 1mm. **(B)** CEJ-ABC linear distances. **(C)** Representative TRAP-stained sections of gingival tissues(top panels, × 40, magnification; scale bar, 0.5 mm, bottom panels, × 100 magnification; scale bar, 200 μm). Arrowheads mark osteoclasts. **(D)** The number of TRAP-positive cells at the ligature site. **(E)** The mRNA expression levels of genes measured using qRT-PCR. **(F)** Representative H&E sections of gingival tissues after ligature treatment (top panels, scar bar, 0.5mm, bottom panels, scar bar, 50μm). **(G)** Inflammatory cells in gingival tissues after ligature placement. **(H)** The mRNA expression levels of inflammatory factors. Data represent 3 independent experiments (*n* = 5). *P <0.05, ** *P* <0.01, *** *P* <0.001, **** *P* <0.0001.

To verify the feasibility of this model, the alveolar bone resorption between M1 and M2 was analyzed using micro-CT. The distances between the CEJ and ABC were measured at four sites for each maxillary molar (both palatal and buccal, distal and mesial) in three-dimensional micro-CT images. The distance between CEJ-ABC was observed to increase significantly and was time dependent in the orthodontic wire treatment group (Figures 1A,B). In contrast, the distance between CEJ-ABC increased slightly and was not time dependent in the *Pg* and *Pg*-LPS treatment groups. (Appendix Figures 2A,B). These results indicated that more severe alveolar bone loss occurred in the orthodontic wire treatment group within a shorter period than in the *Pg* and *Pg*-LPS treatment groups. Further, TRAP staining and quantification also demonstrated that orthodontic wire induced osteoclast infiltration into the interproximal region between the M1 and M2 (Figures 1C,D). The number of osteoclasts in the *Pg* and *Pg*-LPS group was also higher than controls but lower than orthodontic wire group (Appendix Figures 2C,D). It is worth to note that these findings were consistent with our micro-CT imaging results. We also found that steady state levels of *RANKL* mRNA increased while *Ocn* decreased in gingival tissue for the orthodontic wire treatment group. Expression levels of *Alp, RunX2* and *Opg* all exhibited a declining trend over time (Figure 1E). In comparison, the mRNA expression levels for *RANKL* remained unchanged in the *Pg* and *Pg*-LPS groups (Appendix Figures 2E,F). These data demonstrated that the orthodontic wire ligation model resulted in bone resorption and a periodontitis phenotype that was more pronounced than the *Pg* and *Pg*-LPS models.

### Inflammatory response

One key aspect of periodontitis is the infiltration of inflammatory cells to the gingival tissue. As expected, all 3 of our experimental models resulted in enhanced cell infiltration. Interestingly, the greatest numbers of cells were observed in the orthodontic wire ligature group (Figures 1F,G; Appendix Figures 2G,H). Additionally, mRNA expression levels of *Il1*β and *Il6* also increased notably in gingival tissue of orthodontic wire ligature model but increased slightly in the *Pg* and *Pg*-LPS groups (Figure 1H). These results confirmed that a stronger inflammatory response was induced by the orthodontic wire treatment compared to the other models.

### Evaluation of bacterial accumulation

**Figure 2:**
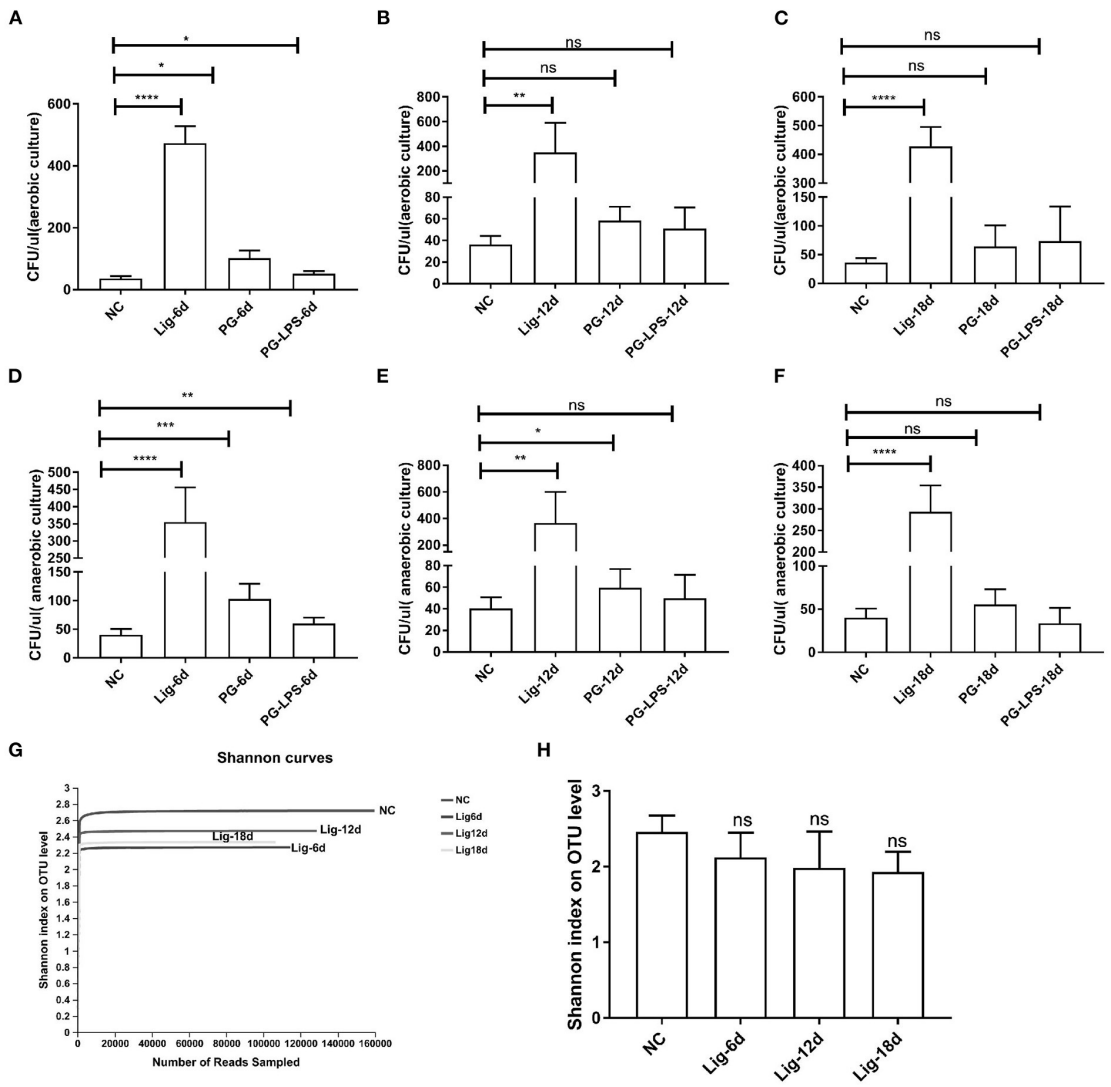
Bacterial accumulation and α diversity of oral microorganisms in the orthodontic wire ligation model. Bacterial colony numbers from oral cavities under **(A–C)** aerobic and **(D–F)** anaerobic conditions. **(G)** Rarefaction curves for the oral bacterial communities. **(H)** Shannon diversity index for the orthodontic wire ligature groups. Data represent 3 independent experiments (*n* = 6). ns, not significant, *P <0.05, ** *P* <0.01, *** *P* <0.001, **** *P* <0.0001.

The transition from health to periodontitis in human correlates with an increased microbial burden. Upon that, we determined the numbers of bacteria in oral cavities of our experimental mice extracted from oral swabs sampling on days 0, 6, 12, and 18 post-ligation, respectively. Results showed that oral bacteria increased significantly under both aerobic and anaerobic culture conditions in the orthodontic wire ligature model, indicating an increase of microbial burden in orthodontic wire ligature-induced periodontitis (Figures 2A–F). We also analyzed bacterial community structure from these samples and generated a total of 1243240 reads with an average number of 41,441 ± 10,088.58 per sample. We identified 32 phyla, 664 genera and 1,043 species. The Shannon α diversity index showed no significant differences between our experimental PD model and the control group. In contrast, the rarefaction curves indicated a decrease in oral bacterial diversity in the orthodontic wire ligature group (Figures 2G,H). Additionally, the oral microbial α-diversity for the Pg and Pg-LPS groups was also unchanged (Appendix Figures 3A–D). Collectively, these results indicated that the orthodontic wire treatment led to accumulation of oral bacteria despite the oral microbial α diversity was not altered during disease progression.

### Bacterial communities composition and β diversity

**Figure 3:**
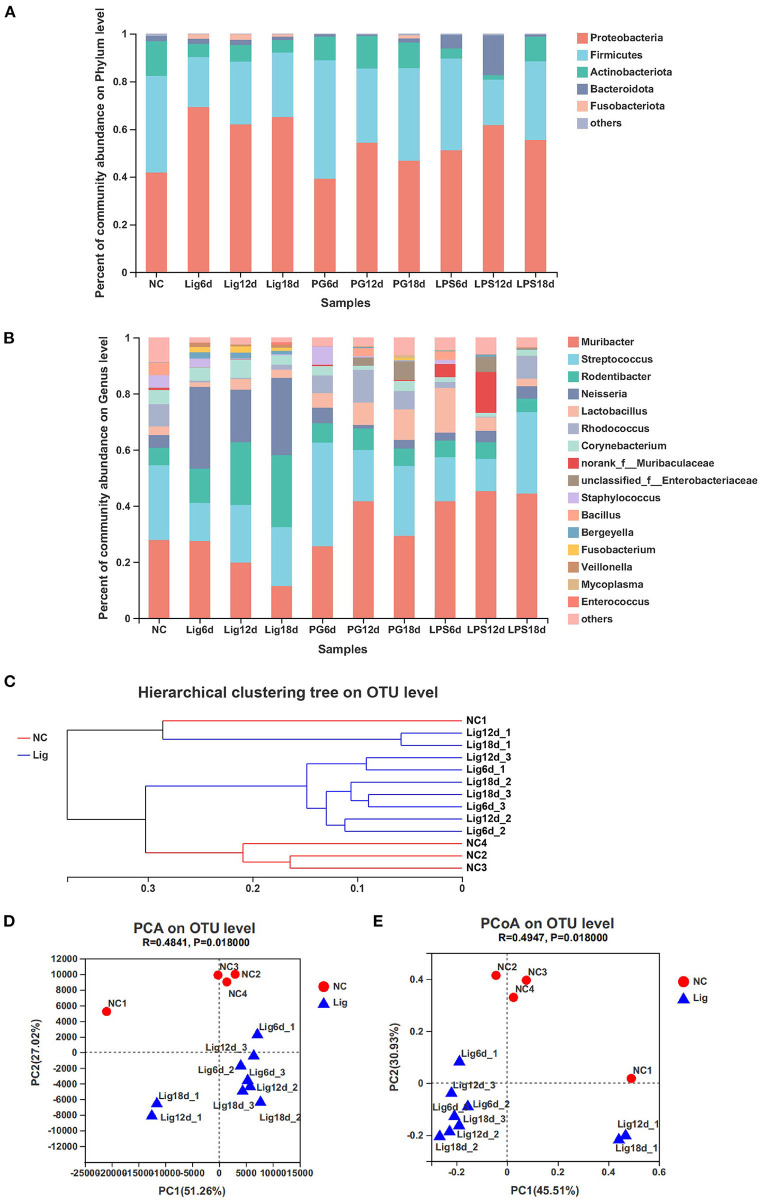
Composition and β diversity of bacterial communities in the orthodontic wire ligation model. **(A,B)** Relative abundance of bacterial phyla and genera identified in mouse oral cavities after ligation treatment. **(C)** Bray-Curtis hierarchical clustering showing the relationship between samples. **(D)** Principal component analysis of bacterial community composition in mouse oral cavities based on ANOSIM. **(E)** PCoA analysis based on Bray-Curtis distancesfor bacterial community compositionsin the samples.

**Figure 4:**
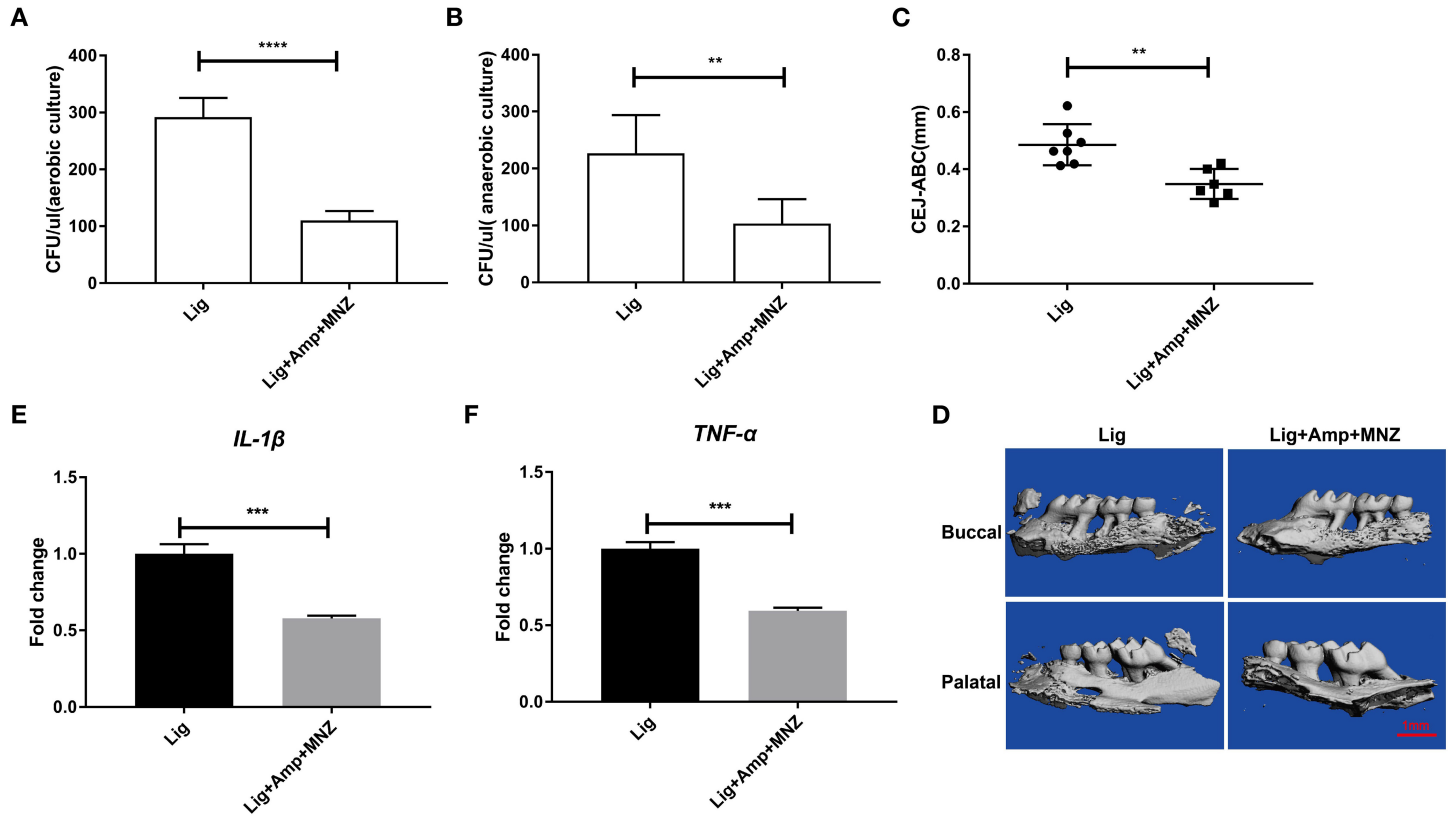
Bone resorption was decreased following antibiotic treatment. Numbers of bacterial colonies in oral cavities of mice under **(A)** aerobic and **(B)** anaerobic conditions. **(C)** The linear distance of CEJ-ABC after antibiotic treatment of mice. **(D)** Representative sagittal 3D images viewed from the buccal side and palatal side of the maxillary molars using Micro-CT, scar bar, 1mm. The mRNA expression levels of IL-1β **(E)** and TNF-α **(F)**. Data represent 3 independent experiments (*n* = 6). ** *P* <0.01, **** *P* <0.0001.

A further analysis of the bacterial communities indicated the presence of 5 dominant phyla including *Firmicutes, Proteobacteria, Actinobacteriota, Bacteroidota* and *Fusobacteria*. The *Proteobacteria* and *Fusobacteria* were the most abundant in the ligature model, suggesting their association with periodontitis. In contrast, *Firmicutes* and *Actinobacterium* were the most abundant in the healthy controls (Figure 3A). These results were also confirmed by qPCR (Appendix Figure 4G). The dominant genera included *Muribacter, Streptococcus, Rodentibacter, Neisseria, Rhodococcus, Bacillus and Staphylococcus*. The relative abundance of *Rodentibacter* and *Neisseria* increased in the ligature group whereas *Muribacter, Streptococcus, Rhodococcus, Bacillus* and *Staphylococcus* decreased (Figure 3B). These results were indicative of an additional change in bacterial community composition during periodontal disease progression. The community structure of ligature group was overall distinct from that of healthy controls, which was apparent in phylogenetic tree analysis (Figure 4C). A principal coordinate analysis (PCoA) based on these Bray-Curtis distances and principal component analysis (PCA) also demonstrated the distinctive composition of the bacterial community between the controls and the ligature group (Figures 3D,E). This revealed that oral bacterial communities converted to a community associated with periodontitis. No significant difference in bacterial community composition was observed for the *Pg* and *Pg*-LPS groups and the healthy controls as determined using phylogenetic trees, PCA and PCoA analyses (Appendix Figures 4A–F). These results suggested that the ligature treatment resulted in the development of oral microbial community dysbiosis.

### Antibiotic treatment reduced alveolar bone resorption

The development of oral microbial community dysbiosis was further explored by treating mice with ampicillin and metronidazole before and during treatments. After antibiotic treatment the oral bacterial loads under both aerobic and anaerobic growth conditions decreased (Figures 4A,B). This was also assessed by measuring alveolar bone resorption *via* the CEJ-ABC distances. The antibiotic treatment decreased the CEJ-ABC distances, indicating that oral bacterial loads were positively associated with alveolar bone resorption (Figures 4C,D). Antibiotic treatment also significantly decreased *Il1*β and *Tnf-*α genes expression (Figures 4E,F). These results indicated that inhibition of bacterial growth by antibiotic administration decreased both inflammation and alveolar bone loss in response to microbial community dysbiosis.

## Discussion

The classical silk ligature model has been used in different animals, including mice, rats, rabbits, dogs, and non-human primates (Weiner et al., 1979; Abe and Hajishengallis, 2013; Glowacki et al., 2013; Kajikawa et al., 2017). Among these experimental animal models, the mouse model has several advantages, such as a wide range of genetically engineered strains, the availability of high-quality immunochemical reagents, low cost for small animals compared with large animals and the wide availability of germ-free mice. Unfortunately, the silk ligature model is not commonly used in mice largely due to technical difficulties in inserting the ligature into the interproximal spaces between the molars. Murine wild-type (WT) molars (M3-M1) have a size range of 0.4–1.2 mm (Häärä et al., 2012), making it difficult to clearly see where to place the ligation. For a long time, various attempts have been conducted to address the operative challenge for silk ligature in mice (Abe and Hajishengallis, 2013; Marchesan et al., 2018; Li et al., 2020). Recently, a device designed by 3D printing technology was constructed allowing easily inserted ligatures between two molars in mice (Marchesan et al., 2018). However, the application of this method is limited by the availability of experiment conditions because 3D printing technology is not yet widespread. Over the past decade, several teams have used orthodontic wire instead of silk for ligature modeling, but all have focused only on inflammatory responses and alveolar bone loss, without considering the impact on oral microbiota balance. In this work, we first improved the approach for periodontitis in mice using stainless steel orthodontic wires. In our preliminary experiments, we found that horizontal orthodontic wires often fail to stay in place and sometimes even fall off during experimental periodontitis and the dislodged or detached orthodontic filament was easy to cause oral mucosal tissue damage. Therefore, we bent the orthodontic wire into a small loop which not only reduced the loss rate of ligation wires, but also avoided mucosal tissue damage. As a result, the success rate of ligation modeling rose to 97–100%. It is worth noting that the wire could potentially apply force on the adjacent teeth and induce tooth movement, resulting the inflammatory response and bone resorption. To reduce this impact, a 0.20 mm diameter soft wire was chosen to replace the traditional 0.25 mm silk wire. In addition, the traditional ligation model requires a tight ligation surrounding the root of the maxillary second molar to prevent the silk from falling off. In our simplified ligature model, a small wire ring is placed gently between two molars. These improvements may to some extent weaken the effect of forces on periodontal tissues. Li's study also confirmed that insertion of orthodontic ligatures would not harm the alveolar bone (Li et al., 2020). Compared to affecting the entire region surrounding the tooth with a traditional ligature model, our simplified ligature model affected a smaller area of the periodontium and better reduced the mechanical compression of ligature on gingival tissue. Consequently, the development of bone loss in our model was not as rapid as that seen in the traditional silk model. Substantial alveolar bone loss took place at day 6 rather than day 3 in our model and most progressive bone absorption occurred from day 6 to 12. This dynamic bone loss is generally consistent with that seen in the silk ligature model (Abe and Hajishengallis, 2013; Hu et al., 2017). Compared with the *Pg* and *Pg*-LPS models, the orthodontic wire ligation model exhibited characteristics of more rapid alveolar bone loss, more osteoclast activation, more periodontal immune cell infiltration and more up-regulated inflammatory cytokines.

In periodontitis, microbial communities induce a dysregulated and destructive host response through an overall mechanism referred to as polymicrobial synergy and dysbiosis (Hajishengallis and Lamont, 2012). Conventional culture-based approaches have identified the bacterial species associated in plaque also known as the “red-complex” bacteria; *P. gingivalis, Tannerella forsythia* and *Treponema denticola*(Socransky et al., 1998). Recently, culture-independent metagenomic and metatranscriptomic studies have extended the list of candidate pathogens to include members of the phyla *Actinobacteria, Firmicutes, Proteobacteria* and *Bacteroidetes* and species in the genera *Prevotella, Desulfobulbus, Synergistes* and others raising estimates for the number of bacterial species found in periodontal tissue to >600 (Dewhirst et al., 2010). Therefore, efforts are now underway to understand oral microbial interactions that may lead to either the maintenance of health or the development of disease by focusing on the interplay between these bacteria in the biofilm communities. Similarly to other polymicrobial diseases, periodontitis has been considered as a microbial-shift disease owing to a well-characterized shift in the microbial communities (species composition and abundance of individual species) that are present during the transition from health to disease. In periodontitis, the rise of newly dominant species, rather than the appearance of new species, such as exogenous pathogens that are absent in health, has been observed (Abusleme et al., 2013). Thus, species or genera that dominate disease-associated polymicrobial communities are also found in health but at markedly reduced relative abundance. This is predicted by the ecological plaque hypothesis according to which changes in environmental conditions may favor the outgrowth of pathogens beyond a threshold that can instigate periodontitis (Marsh, 2003). This concept gained experimental support through animal model studies demonstrating that anti-inflammatory treatments not only inhibit periodontitis in mice and rats but also diminish the periodontal bacterial burden and reverse dysbiosis (Hajishengallis et al., 2011; Lee et al., 2016). Consistent with these results, we found an obvious microbiota dysbiosis in our orthodontic wire ligation mouse model. The whole genome sequencing approach using 16S rDNA demonstrated that the dominant phyla in the oral cavity of mice were primarily *Firmicutes, Proteobacteria, Actinobacteriota, Bacteroidota* and *Fusobacteria*, which is consistent with the dominant phyla found in humans. The microbial community shift during experimental mouse periodontitis was characterized by an increase in *Proteobacteria* and *Fusobacteria* and a decrease in *Firmicutes* and *Actinobacteria*. Furthermore, PCA and PCoA analysis demonstrated that the oral microbial communities in the ligation model were significantly different from that of the controls, indicating an oral microbiota community shift from a symbiotic to a dysbiotic microbial community structure. In contrast, this type of shift for the *Pg* and *Pg*-LPS groups was merely slight compared with controls. However, the shifts we found in our mice were inconsistent with those seen in human periodontitis. This was most likely due to the fact mice carry their endogenous bacteria, which differs from the case for humans. This suggests that our mouse model could be used to explore human bacterial colonization and induction of host responses *in vivo* by inserting a ligature into germ-free mice and inoculating these mice with human oral bacteria. In addition, after treatment with ampicillin and metronidazole, the alveolar bone absorption was significantly enhanced and the oral bacterial load decreased in the periodontitis mice. This suggested that the alterations in the oral microbial community were closely related to periodontitis pathogenesis.

In conclusion, we improved a simplified and effective approach for initiating a mouse model of periodontitis, by which progressive alveolar bone loss and periodontal inflammation occurred following orthodontic ligature wire placement. Furthermore, the orthodontic wire ligation model can mimic polymicrobial communities characterized by polymicrobial synergy and dysbiosis. Therefore, our model could be suitable for further study of pathogenesis of periodontal disease and exploring corresponding treatment strategies.

## Data availability statement

The original contributions presented in the study are included in the article/Supplementary materials, further inquiries can be directed to the corresponding author/s.

## Ethics statement

The animal study was reviewed and approved by the Institutional Animal Care and Use Committee's guidelines at Chongqing Medical University.

## Author contributions

RA contributed to conception and design, data acquisition and analysis, drafted, and critically revised the manuscript. DL and LS contributed to data acquisition and analysis, and critically revised the manuscript. XZ and ZD contributed to conception and critically revised the manuscript. YZ contributed to data acquisition and analysis and critically revised the manuscript. YH contributed to conception and design, data interpretation, and drafted and critically revised the manuscript. All authors gave final approval and agree to be accountable for all aspects of the work.

## Funding

This work was supported by Chongqing Research Program of Basic Research and Frontier Technology (No. cstc2018jcyjAX0257).

## Conflict of interest

The authors declare that the research was conducted in the absence of any commercial or financial relationships that could be construed as a potential conflict of interest.

## Publisher's note

All claims expressed in this article are solely those of the authors and do not necessarily represent those of their affiliated organizations, or those of the publisher, the editors and the reviewers. Any product that may be evaluated in this article, or claim that may be made by its manufacturer, is not guaranteed or endorsed by the publisher.

## Supplementary material

Appendix Figure 1 The orthodontic wire ligature model. **(A)** Crown scissors, curved tweezers and 0.2 mm stainless steel orthodontic wire used in the process. **(B)** Detailed procedures of orthodontic wire insertion. Step 1: insert orthodontic wire from maxillary side to buccal side into the gap between M1 and M2. Step 2: bend the orthodontic wire on the buccal side toward the direction indicated by the arrow. Step 3: anastomose the orthodontic wire to form a ring. Step 4: cut redundant orthodontic wire with crown scissors.

Appendix Figure 2 Alveolar bone resorption and inflammation in the PG and PG-LPS groups. Representative sagittal 3D images viewed from the buccal side and palatal side of the maxillary molars by Micro-CT image and the linear distance of CEJ-ABC for **(A)** PG and **(B)** PG-LPS models. Representative TRAP-stained sections and numbers of TRAP+ cells in alveolar bone for the **(C)** PG and **(D)** PG-LPS models. The mRNA expression levels of genes related to osteogenesis was measured using RT-qPCR for **(E)** PG and **(F)** PG-LPS models. Representative H&E sections of gingival tissues for **(G)** PG and **(H)** PG-LPS models. Data represent 3 independent experiments (n = 3). ns, not significant, ^*^*P* <0.05, ^**^ *P* <0.01, ^***^ *P* <0.001, ^****^ *P* <0.0001.

Appendix Figure 3 α diversity of oral microorganisms. Rarefaction curves for the **(A)** PG and **(B)** PG-LPS groups. Shannon diversity index of oral bacterial community for the **(C)** PG and **(D)** PG-LPS groups. ns, not significant, ^*^*P* <0.05.

Appendix Figure 4 β diversity of bacterial communities in the PG and PG-LPS groups. Hierarchical clustering based on the Bray-Curtis method for **(A)** PG and **(B)** PG-LPS models. PCA of bacterial community composition in mouse oral cavities based on ANOSIM for **(C)** PG and **(D)** PG-LPS models. PCoA analysis based on the Bray-Curtis distances for bacterial community compositions in the samples for **(E)** PG and **(F)** PG-LPS models. **(G)** Bar graphs represent the changes in the levels of 16S rRNA for each bacterial phylum following orthodontic wire placement normalized to control mice. ns, not significant, ^*^*P* <0.05.
