# Supplementary figures and images for: Periodontitis induced by orthodontic wire ligature drives oral microflora dysbiosis and aggravates alveolar bone loss in an improved murine model
Source: Front Microbiol. 2022 Sep 8;13:875091. doi: 10.3389/fmicb.2022.875091 (PMC9493320; doi:10.3389/fmicb.2022.875091)

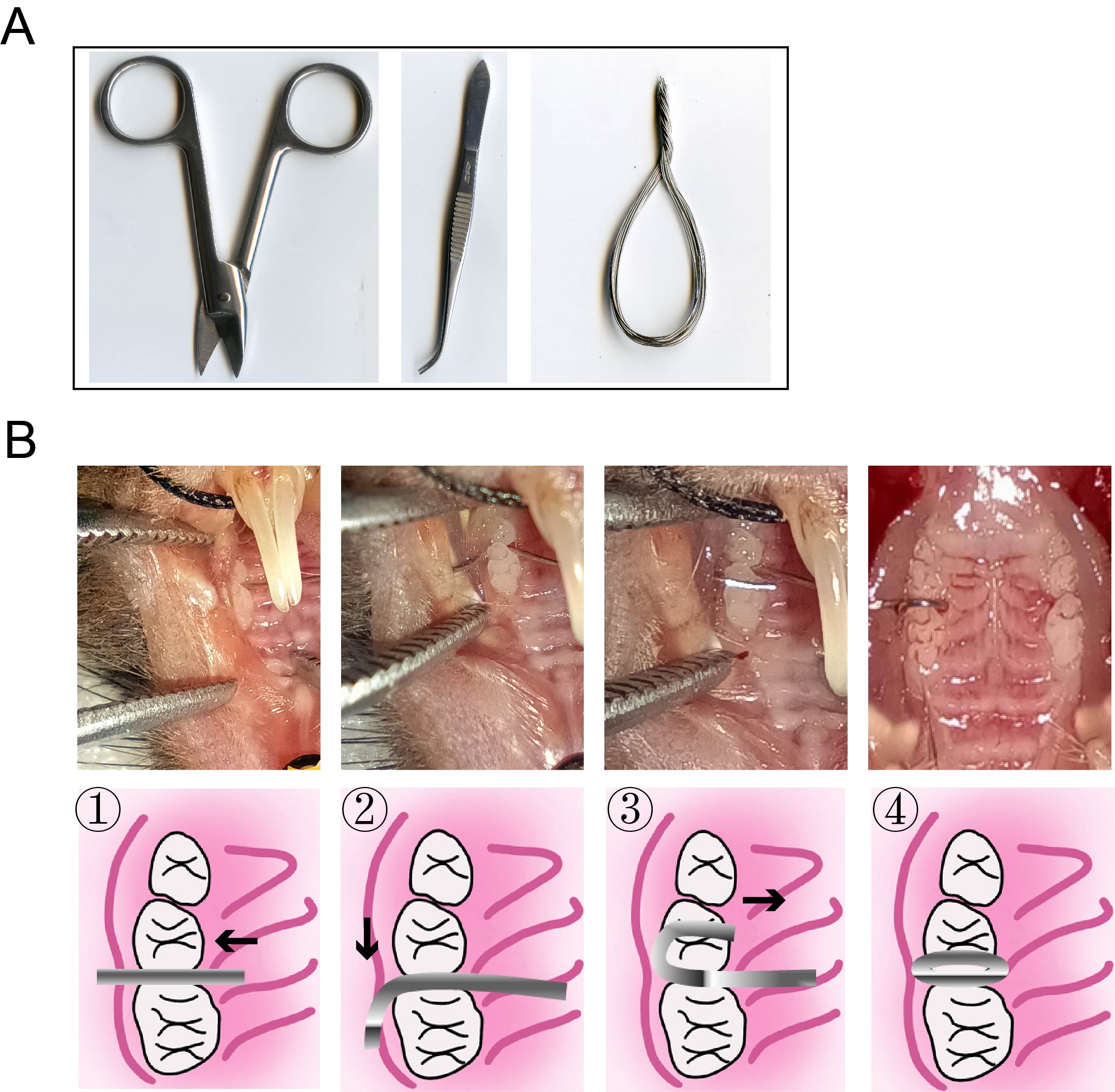

Supplement: Appendix Figure 1 — The orthodontic wire ligature model. (A) Crown scissors, curved tweezers and 0.2 mm stainless steel orthodontic wire used in the process. (B) Detailed procedures of orthodontic wire insertion. Step 1: insert orthodontic wire from maxillary side to buccal side into the gap between M1 and M2. Step 2: bend the orthodontic wire on the buccal side toward the direction indicated by the arrow. Step 3: anastomose the orthodontic wire to form a ring. Step 4: cut redundant orthodontic wire with crown scissors. [file Image_1.JPEG]

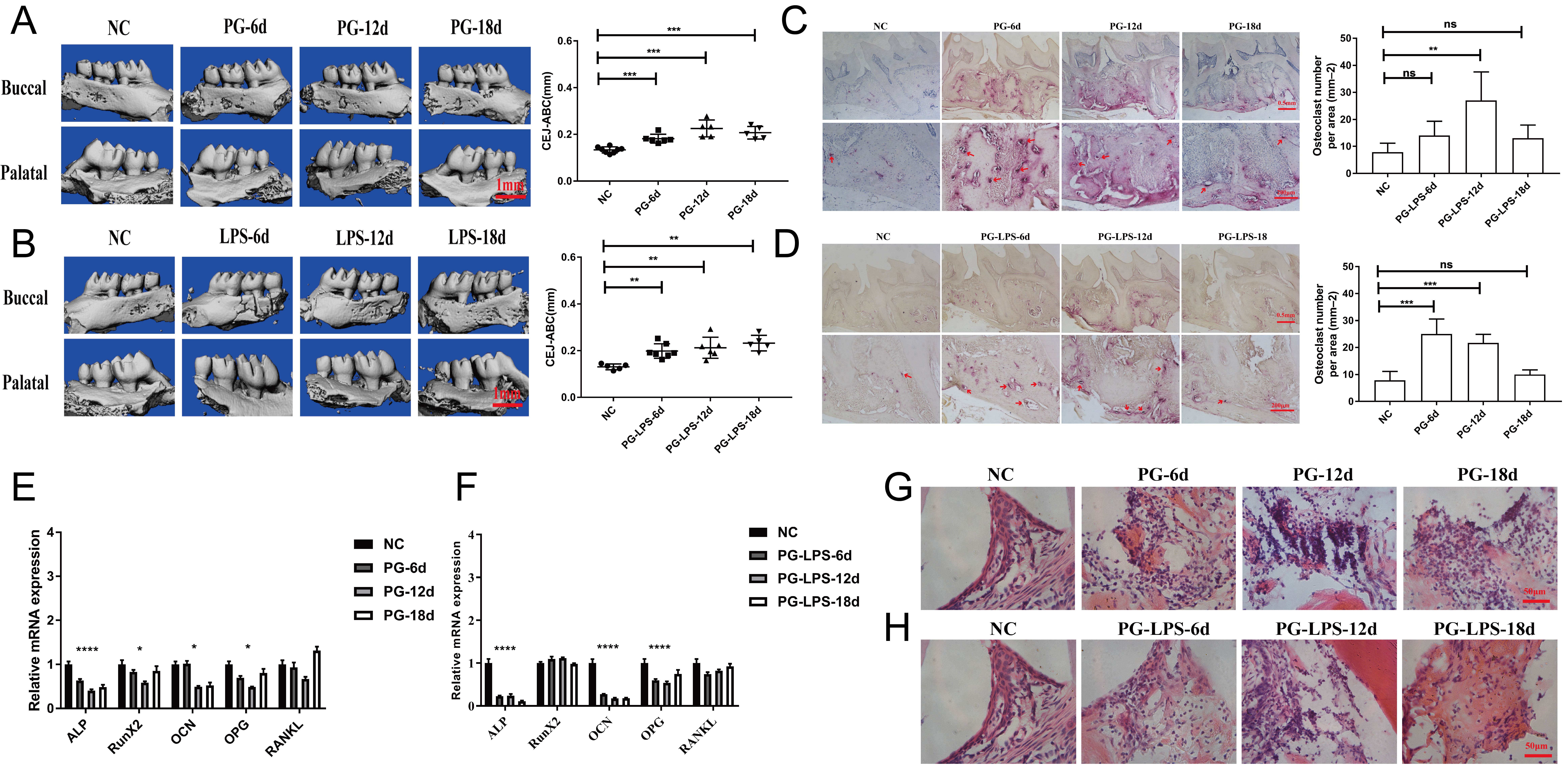

Supplement: Appendix Figure 2 — Alveolar bone resorption and inflammation in the PG and PG-LPS groups. Representative sagittal 3D images viewed from the buccal side and palatal side of the maxillary molars by Micro-CT image and the linear distance of CEJ-ABC for (A) PG and (B) PG-LPS models. Representative TRAP-stained sections and numbers of TRAP+ cells in alveolar bone for the (C) PG and (D) PG-LPS models. The mRNA expression levels of genes related to osteogenesis was measured using RT-qPCR for (E) PG and (F) PG-LPS models. Representative H&E sections of gingival tissues for (G) PG and (H) PG-LPS models. Data represent 3 independent experiments (n = 3). ns, not significant, *P <0.05, ** P <0.01, *** P <0.001, **** P <0.0001. [file Image_2.JPEG]

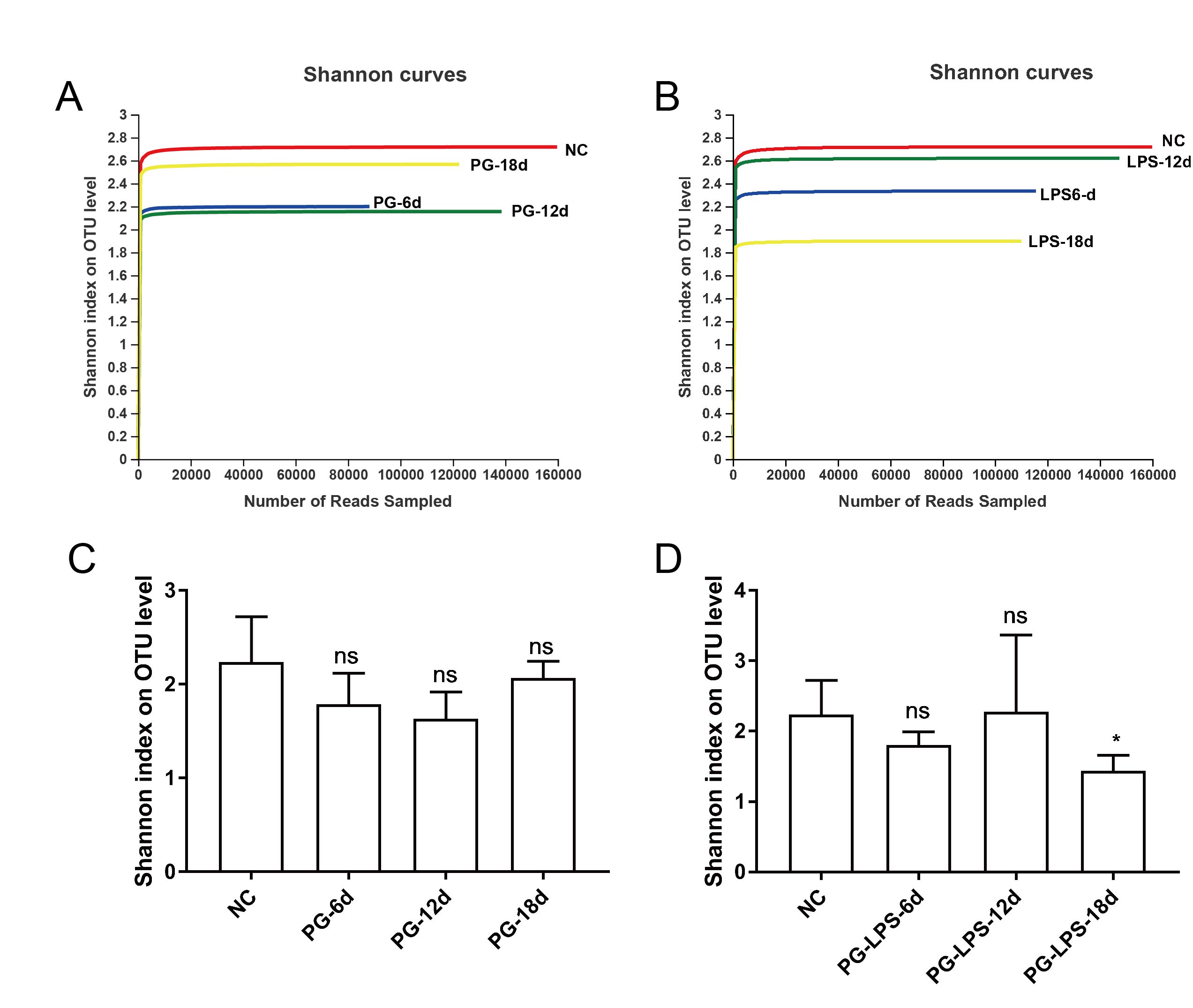

Supplement: Appendix Figure 3 — α diversity of oral microorganisms. Rarefaction curves for the (A) PG and (B) PG-LPS groups. Shannon diversity index of oral bacterial community for the (C) PG and (D) PG-LPS groups. ns, not significant, *P <0.05. [file Image_3.JPEG]

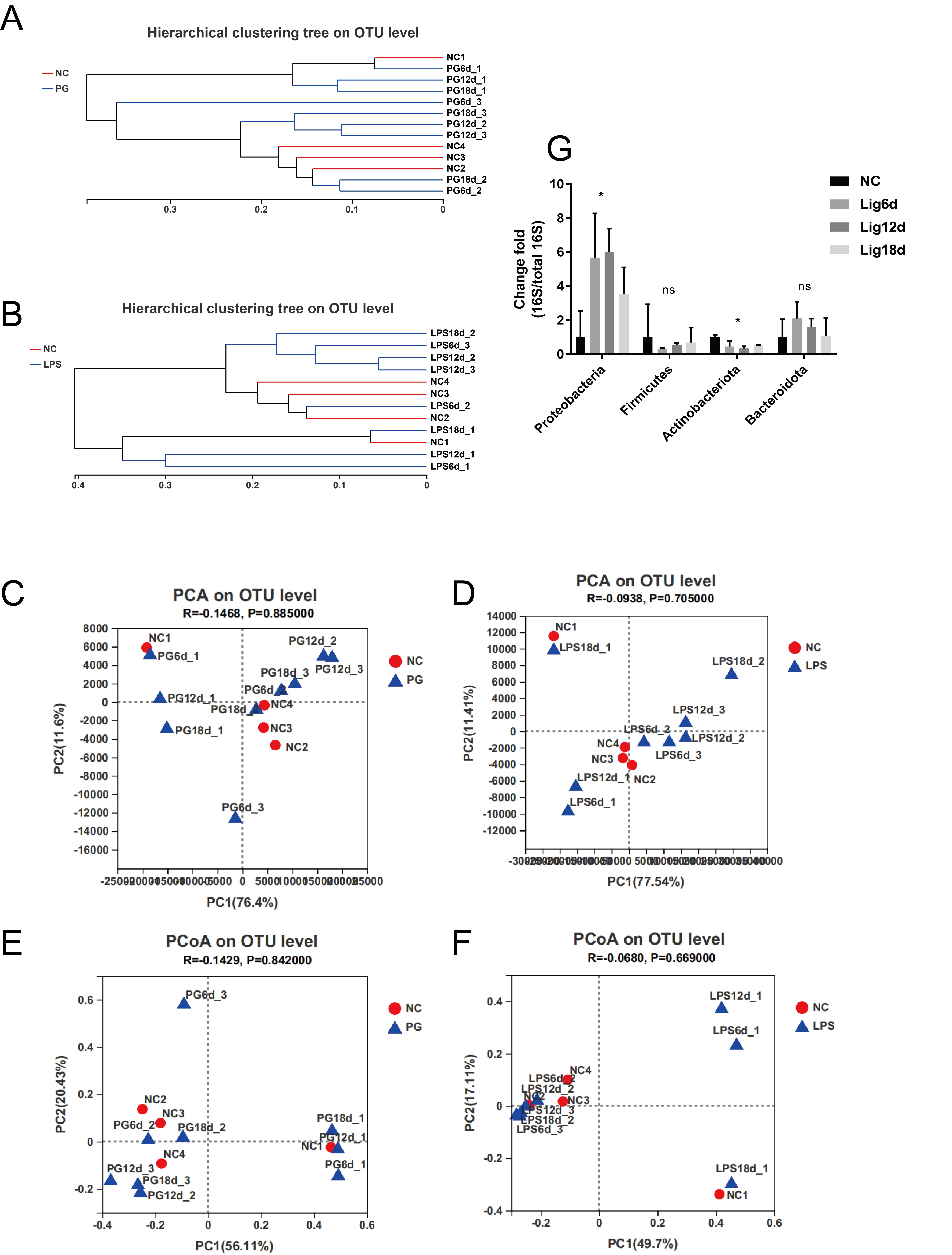

Supplement: Appendix Figure 4 — β diversity of bacterial communities in the PG and PG-LPS groups. Hierarchical clustering based on the Bray-Curtis method for (A) PG and (B) PG-LPS models. PCA of bacterial community composition in mouse oral cavities based on ANOSIM for (C) PG and (D) PG-LPS models. PCoA analysis based on the Bray-Curtis distances for bacterial community compositions in the samples for (E) PG and (F) PG-LPS models. (G) Bar graphs represent the changes in the levels of 16S rRNA for each bacterial phylum following orthodontic wire placement normalized to control mice. ns, not significant, *P <0.05. [file Image_4.JPEG]
